# Polyphenolic Composition of *Rosa canina*, *Rosa sempervivens* and *Pyrocantha coccinea* Extracts and Assessment of Their Antioxidant Activity in Human Endothelial Cells

**DOI:** 10.3390/antiox8040092

**Published:** 2019-04-06

**Authors:** Efthalia Kerasioti, Anna Apostolou, Ioannis Kafantaris, Konstantinos Chronis, Eleana Kokka, Christina Dimitriadou, Evangelia N. Tzanetou, Alexandros Priftis, Sofia D. Koulocheri, Serkos A. Haroutounian, Demetrios Kouretas, Dimitrios Stagos

**Affiliations:** 1 Department of Biochemistry and Biotechnology, University of Thessaly, Viopolis, 41500 Larissa, Greece; e-f-thalia@hotmail.com (E.K.); kafantarisioannis@gmail.com (I.K.); kochronis@uth.gr (K.C.); elkokka@uth.gr (E.K.); xristina_211@hotmail.com (C.D.); priftis@uth.gr (A.P.); dkouret@uth.gr (D.K.); 2 Laboratory of Nutritional Physiology and Feeding, Agricultural University of Athens, Iera Odos 75, 11855 Athens, Greece; annapost21@msn.com (A.A.); eyatza@gmail.com (E.N.T.); skoul@aua.gr (S.D.K.); sehar@aua.gr (S.A.H.); 3 Department of Pesticides Control and Phytopharmacy, Benaki Phytopathological Institute, 8 St. Delta Street, Kifissia, 14561 Athens, Greece

**Keywords:** Rosa canina, Rosa sempervivens, Pyrocantha coccinea, polyphenols, antioxidants, endothelial cells, glutathione

## Abstract

The aim of the present study was the investigation of the antioxidant activity of plant extracts from *Rosa canina, Rosa sempervivens* and *Pyrocantha coccinea*. The results showed that the bioactive compounds found at higher concentrations were in the *R. canina* extract: hyperoside, astragalin, rutin, (+)-catechin and (-)-epicatechin; in the *R. sempervirens* extract: quinic acid, (+)-catechin, (−)-epicatechin, astragalin and hyperoside; and in the *P. coccinea* extract: hyperoside, rutin, (−)-epicatechin, (+)-catechin, astragalin, vanillin, syringic acid and chlorogenic acid. The total polyphenolic content was 290.00, 267.67 and 226.93 mg Gallic Acid Equivalent (GAE)/g dw, and the total flavonoid content 118.56, 65.78 and 99.16 mg Catechin Equivalent (CE)/g dw for *R. caninna, R. sempervirens* and *P. coccinea* extracts, respectively. The extracts exhibited radical scavenging activity in DPPH and 2,2’-azino-bis(3-ethylbenzothiazoline-6-sulphonic acid) (ABTS)•^+^ assays and protection from ROO•-induced DNA damage in the following potency order: *R. canina* > *R. sempervirens* > *P. coccinea*. Finally, treatment with *R. canina* and *P. coccinea* extract significantly increased the levels of the antioxidant molecule glutathione, while *R. canina* extract significantly decreased Reactive Oxygen Species (ROS) in endothelial cells. The results herein indicated that the *R. canina* extract in particular may be used for developing food supplements or biofunctional foods for the prevention of oxidative stress-induced pathological conditions of endothelium.

## 1. Introduction

It is well known that within living organisms, the reactive oxygen species (ROS) are produced endogenously under physiological processes such as metabolism and inflammation [1,2]. Low ROS levels are needed for the progression of several basic biological processes including cellular proliferation and differentiation [2,3]. However, excessive intracellular concentration of ROS can lead to oxidative stress, a pathological condition associated with the development of various of diseases including cancer, diabetes, rheumatoid arthritis and other degenerative diseases in humans [4,5]. Oxidative stress causes healthy cells of the body to lose their function and structure as a consequence of ROS interaction with proteins, lipids and DNA [6].

One of the tissues that is especially susceptible to oxidative stress is vascular endothelium, a critical regulator of vascular homeostasis. Endothelial cell injury or dysfunction by ROS can be both a cause and consequence of many vascular complications including atherosclerosis, thrombosis and cardiovascular diseases [7,8]. In particular, vascular endothelium cells are susceptible to ROS damage, since ROS derived from different tissues circulate in the bloodstream and can interact directly with endothelial cells in the inner wall of blood vessels [9,10]. In addition, oxidative stress may cause damage to the endothelium through leukocyte adhesion [11].

However, human organism possesses several antioxidant mechanisms to counteract the overproduction and harmful effects of ROS [12]. The antioxidant protective mechanisms act in order to keep a balance between free radical production and scavenging. These antioxidants are either produced endogenously or obtained through diet, especially from plant foods [13]. The antioxidant proprieties of plant foods are mainly attributed to polyphenols, a large group of secondary metabolites, that possess antiradical activities due to their phenolic hydroxyls acting as reducing agents, metal ion chelators, antioxidant enzymes activators, and oxidases inhibitors [14,15]. Thus, there is currently a great interest in natural sources of antioxidants in order to improve the redox status and protect the organism from the detrimental effects of oxidative stress. 

Many plant species of the *Rosaceae* family are considered to be of high importance because of their use in various food preparations as jam, tea and beverages. The *Rosa* genus of *Rosaceae* family consists of approximately 200 species located mainly in the Northern hemisphere in rainy areas and deserts [16]. *Rosa* species produce rose hips, a pseudocarp or a fruit. A number of studies have shown that rose hips demonstrate a great variety of bioactivities such as antioxidant, anticancer, anti-inflammatory and anti-obesity activities [17,18,19,20]. Wild fruits of *Rosa canina* are rich in vitamin C and are used for the prevention and/or treatment of many diseases including diabetes, flu, arthritis, inflammations, pain and diarrhea [17,18,21]. *Rosa sempervirens,* the evergreen rose, is a representative member of the *Rosa* species. It is a thorny, climbing rose characterized by long branches with few or no prickles. Moreover, it is an important source of vitamin C, carotenoids, polyphenols, organic acids and tocopherols [22]. *Pyracantha coccinea* of the genus *Pyracantha* of the *Rosaceae* family is a plant growing from South Europe to South-East Asia. Its fruits are known for their rich content in fatty acids, polyphenolic compounds, phytosterols and vitamins. Thus, they have been used in traditional medicines for cardiac and diuretic properties [23].

The aim of the present study was to investigate the antioxidant properties of polyphenolic extracts derived from the following three wild *Rosaceae* species of Greece; *Pyracantha coccinea* (fruit extract), *Rosa sempervirens* (fruit extract) and *Rosa canina* (fruit extract). The extracts were initially examined for their free radical scavenging activity against DPPH• and 2,2’-azino-bis(3-ethylbenzothiazoline-6-sulphonic acid) (ABTS)•^+^ radicals and for their protective effects against peroxyl (ROO•) radical-induced DNA damage. Moreover, for the first time, the ability of these extracts to enhance the antioxidant defense in human endothelial cells was assessed. As mentioned above, the oxidative stress-induced endothelium damage is a crucial etiological factor for cardiovascular diseases, and so the identification of compounds that could protect from such damage is important.

## 2. Materials and Methods

### 2.1. Plant Material and Preparation of the Extracts

Fresh plant material of the investigated *Rosaceae* family plants *Rosa sempervirens* (fruit), *Rosa canina* (fruit) and *Pyracantha coccinea* (fruit) were collected during early summer of 2014, from respective wild plants colonies that grow on Parnitha mountain (Attica Region, Greece). A voucher specimen for each sampling material has been deposited in the herbarium of the Agricultural University of Athens (Athens, Greece). 

After air-drying, 500 g of each plant material were crushed, homogenized with a blender and lyophilized to provide a powder, which was stirred in darkness with 1 L of methanol (HPLC-analytical grade) for 48 h. Then, the solution was filtered and the solid was re-extracted twice following the same procedure. The combined methanolic extracts (3 L in total) were evaporated to dryness under reduced pressure to provide crude extracts, which were further elaborated for the assessment of their chemical content and bioactivities.

### 2.2. Determination of Extracts’ Polyphenolic Content Using HPLC and Ultra Performance Liquid Chromatography–Tandem Mass Spectrometer (UPLC-MS-MS) Analysis

The chemical composition of the extracts was determined using HPLC analysis performed on a Hewlett Packard HP1100 (Hewlett Packard, Palo Alto, CA, USA) equipped with an Agilent 1100 diode-array detector (Agilent Technologies, Santa Clara, CA, USA) (measuring absorbance over the full wavelength range during the entire run), a quaternary pump, degasser and coupled to HP ChemStation utilizing the manufacturer’s 5.01 software package system (Hewlett Packard, Palo Alto, CA, USA). 

The column used was a Zorbax Eclipse Plus C18, 5 μm, 150 × 4.6 mm i.d. chromatographic column (Agilent Technologies, Santa Clara, CA, USA), connected with a guard column of the same material (8 × 4 mm). Injection was by means of a Rheodyne injection valve (model 7725I) with a 20 μL fixed loop. For the chromatographic analyses HPLC-grade water was prepared using a Milli-Q system (Merck Millipore, Burlington, MA, USA), whereas all HPLC solvents (except acetonitrile) were filtered prior to use through cellulose acetate membranes of 0.45 μm pore size. 

The mobile phase was composed of a gradient system of 0.3% acetic acid in water (A) and acetonitrile (B). The flow rate was maintained at 1 mL/min and the column gradient elution program consisted of: 25% B (0 min), 25% B (5 min), 30% B (10 min), 40% B (15 min), 50% B (20 min) and 70% B (30 min) where it remained for additional 5 min, and returned during 2 min to initial conditions, where it stayed for additional 2 min. This routine was followed by a 15 min equilibration period with the zero-time mixture prior to injection of the next sample. Prolonged runtimes (extended until 100 min) were also applied to determine constituents that elute after the 35 min (betulinic and ursolic acids). Peaks were identified by comparing their retention times and UV–vis spectra with the reference compounds, and data were quantitated using the corresponding curves of the reference compounds as standards (Extrasynthese, Genay Cedec, France; Sigma-Aldrich, St. Louis, MO, USA; Alfa Aesar, Haverhill, MA, USA; Fluka, Buchs, Switzerland). Confirmatory UPLC-MS-MS analysis was carried out on a Thermo Scientific Ultra High Performance Liquid Chromatography system (Waltham, MA, USA) coupled to a TSQ Quantum Vantage (Thermo Fischer Scientific, San Jose, CA, USA) triple quadrupole mass spectrometer. Mass spectrometric analysis was conducted using a heated electrospray ionization (HESI) operating in two complementary modes (positive and negative mode). Selected ion monitoring (SIM) mode was primarily used to confirm the presence of analytes. In selected cases of compounds, tandem mass spectrometry (MS/MS) utilizing the multiple reaction monitoring mode (MRM) was employed for additional confirmation [quinic acid: parent ion, *m/z* 191 (*m/z* 85, 93 product ions), rutin: parent ion, *m/z* 609 (*m/z* 300, 271 product ions), quercetin: parent ion, *m/z* 300.9 (*m/z* 179, 151 product ions), quercitrin: parent ion, *m/z* 447.1 (*m/z* 301, 300, 271 product ions)]. The working conditions were the following: spray voltage 4.2 kV; vaporizer and capillary temperatures 280 and 260 °C respectively, while sheath and auxiliary gas at 60 and 40 arbitrary units respectively. The LC separation was achieved on a Hypersil Gold. 3 μm. 150 × 3 mm i.d. chromatographic column (Thermo Fischer Scientific, San Jose, CA, USA). The mobile phase and the gradient system were identical to the above mentioned for the HPLC-UV analysis, using a flow rate of 0.3 mL/min. Water, acetonitrile, and acetic acid were purchased from Merck (Darmstadt, Germany) and all were LC-MS grade. PTFE filters (0.45 μm) were obtained from Macherey-Nagel, Duren, Germany. All measurements were repeated three times.

### 2.3. Analytical Method Validation

With respect to the analytical method validation part, the linearity for all analytes was determined within the ranges of 10–1000 ng/mL (using matrix matched calibration standards), demonstrating acceptable correlation coefficient values (*r^2^* ≥ 0.99). Recovery of the investigated compounds (as a criterion of the trueness of the method) was evaluated at two concentration levels (40 and 200 ng/mL) by the addition of mixed solutions of the standards into the respective extract and fell within the acceptable range of 70%−120%. Precision values were always acceptable with percent Relative Standard Deviation (RSD%) < 15%. 

### 2.4. Assessment of the Total Polyphenolic Content of the Extracts

The total polyphenolic content (TPC) of the extracts was evaluated by the Folin-Ciocalteu method as described previously [24]. Briefly, 20 μL of the extract were added to a tube containing 1 mL of deionized water. 100 μL of Folin-Ciocalteu reagent was added to the reaction mixture, followed by incubation for 3 min at room temperature. Afterwards, 280 μL of 25% *w/v* sodium carbonate solution and 600 μL of deionized water were added to the mixture. Following 1 h incubation at room temperature in the dark, the absorbance was measured at 765 nm versus a blank containing Folin-Ciocalteu reagent and distilled water without the extract. The measurement of absorbance was conducted on a Hitachi U-1900 ratio beam spectrophotometer (Tokyo, Japan). The optical density of the sample (20 μL) in 25% *w/v* solution of sodium carbonate (280 μL) and distilled water (1.7 mL) at 765 nm was also measured. TPC was determined by a standard curve of absorbance values in correlation with standard concentrations (50–1500 μg/mL) of gallic acid. The total polyphenolic content was expressed as mg of gallic acid equivalents (GAE) per gram of dried weight (dw) of extract. 

### 2.5. Total Flavonoid Content of the Extracts

The total flavonoid content (TFC) of the extracts was evaluated as described previously with minor changes [25]. In particular, 1 mL of the methanolic extract was added into a 10 mL flask containing 4 mL of deionized water. Then, 0.3 mL of sodium nitrite (5%) were added to this mixture and allowed to stand for 5 min at room temperature. Then, 0.3 mL of AlCl_3_·6H_2_O (10% ethanolic) was added, the mixture was allowed to stand for 1 min at room temperature and 2 mL of potassium hydroxide (1 M) was added. The solution was diluted to 10 mL with the addition of deionized water and the absorbance of the solution versus a blank at 510 nm was measured immediately. Flavonoid content was expressed as mg of catechin equivalents (CE) per gram of dry weight of extract by using a standard curve (absorbance versus concentration) prepared from authentic catechin sample. 

### 2.6. Free Radical Scavenging Activity

Free radical scavenging activity of the extracts was evaluated using the 2,2-diphenyl-picrylhydrazyl (DPPH•) and 2,2’-azino-bis(3-ethylbenzthiazoline-6-sulfonic acid) (ABTS•^+^) radical scavenging assays [26,27]. Regarding the DPPH• assay, 1.0 mL of freshly-made methanolic solution of DPPH• radical (100 μM) was mixed with the tested extract solution at different concentrations. The contents were vigorously mixed, incubated at room temperature in the dark for 20 min and the absorbance was measured at 517 nm. The measurement was conducted on a Hitachi U-1900 ratio beam spectrophotometer (Tokyo, Japan). In each experiment, the tested sample alone in methanol was used as blank and DPPH• alone in methanol was used as control. ABTS•^+^ radical scavenging activity of the extracts was determined as described previously [27] with slight modifications. Briefly, ABTS•^+^ radical was produced by mixing 2 mM ABTS with 30 μM H_2_O_2_ and 6 μM horseradish peroxidase (HRP) enzyme in 1 mL of distilled water. The solution was vigorously mixed, incubated at room temperature in the dark for 45 min until ABTS•^+^ radical formation. Then, 10 μL of different extract concentrations were added in the reaction mixture and the absorbance at 730 nm was read. The measurement was conducted on a Hitachi U-1900 ratio beam spectrophotometer (Tokyo, Japan). In each experiment, the tested sample in distilled water containing ABTS and H_2_O_2_ was used as blank, and the ABTS•^+^ radical solution with 10 μL H_2_O was used as control. 

The percentage of radical scavenging capacity (RSC) of the tested extracts, for both assays was calculated according to the following equation:

Radical scavenging capacity (%) = [(A_control_ − A_sample_)/ A_control_] × 100

where A_control_ and A_sample_ are the absorbance values of the control and the tested sample respectively. Moreover, in order to compare the radical scavenging efficiency of the extracts, the IC_50_ value showing the concentration that caused 50% scavenging of DPPH• and ABTS•^+^ radical was calculated from the graph plotted RSC percentage against extract concentration. All experiments were carried out in triplicate and at least on two separate occasions.

### 2.7. Peroxyl Radical-Induced DNA Plasmid Strand Cleavage

The peroxyl radical-induced DNA plasmid strand cleavage assay was performed as described previously [28]. In brief, peroxyl radicals (ROO•) were produced from thermal decomposition of 2,2’-azobis(2-amidinopropane hydrochloride) (AAPH). The reaction mixture (10 μL) containing 1 μg Bluescript-SK+ plasmid DNA, 2.5 mM AAPH in phosphate-buffered saline (PBS) and the tested extract at different concentrations was incubated in the dark for 45 min at 37 °C. Then, the reaction was stopped by the addition of 3 μL loading buffer (0.25% bromophenol blue and 30% glycerol). After analyzing the DNA samples by agarose gel electrophoresis, they were photographed and analyzed using the Alpha Innotech Multi Image (ProteinSimple, San Jose CA, USA). In addition, plasmid DNA was treated with each extract alone at the highest concentration used in the assay in order to test their effects on plasmid DNA conformation. The percentage of the protective activity of the tested extracts from ROO•-induced DNA strand breakage was calculated using the following formula:

% Inhibition = [(S − S_o_) / (S_control_ − S_o_)] × 100

where S_control_ is the percentage of supercoiled DNA in the negative control sample (plasmid DNA alone), S_o_ is the percentage of supercoiled plasmid DNA in the positive control sample (without tested extracts but in the presence of the radical initiating factor) and S is the percentage of supercoiled plasmid DNA in the sample with the tested extracts and the radical initiating factor. Moreover, IC_50_ values showing the concentration that inhibited the AAPH-induced relaxation by 50% were calculated from the graph plotted percentage inhibition against extract concentration. At least two independent experiments in triplicate were performed for each tested compound.

### 2.8. Cell Culture Conditions

As previously described [29], human endothelial EA.hy926 cells were cultured in normal Dulbecco’s modified Eagle’s medium (DMEM) in plastic disposable tissue culture flasks at 37 °C in 5% carbon dioxide.

### 2.9. XTT Cell Viability Assay

For examining the extracts’ antioxidant activity in endothelial cells, non-cytotoxic concentrations were used. For selecting these concentrations, extracts’ cytotoxicity in endothelial cells was checked using the XTT cell viability assay kit (Roche, Switzerland) as previously described [30]. Briefly, EA.hy926 cells were seeded into a 96-well plate with 1 × 10^4^ cells per well in DMEM medium. After 24 h incubation, the cells were treated with different concentrations of the extracts in serum-free DMEM medium for 24 h. Then, 50 μL of XTT test solution was added to each well. After 4 h of incubation, absorbance was measured at 450 nm and also at 630 nm as a reference wavelength in a Bio-Tek ELx800 microplate reader (Winooski, VT, USA). The negative control was DMEM serum-free medium. The absorbance values of the control and samples were used for calculating the percentage inhibition of cell growth caused by the extract treatment. All experiments were carried out in triplicate and on two separate occasions.

### 2.10. Treatment of EA.hy926 Cells with the Extracts

The extracts from *R. sempervirens*, *R. canina* and *P. coccinea* were examined for their antioxidant capacity in endothelial EA.hy926 cells. The cells were cultured in flasks for 24 h. Afterwards the medium was replaced with a serum-free medium containing the tested extracts at non-cytotoxic concentrations. The cells were treated with the extracts for 24 h, and then they were trypsinized, collected and centrifuged twice at 300× *g* for 10 min at 5 °C. At the end of the first centrifugation, the supernatant fluid was discarded and the cellular pellet was resuspended in PBS. After the second centrifugation, the cell pellet was collected and used for measuring the glutathione (GSH) and ROS levels.

### 2.11. Assessment of GSH and ROS Levels by Flow Cytometry Analysis in Endothelial Cells

The GSH and ROS levels in EA.hy926 cells were assessed using mercury orange and DCF-DA, respectively, as described previously [31,32]. In brief, the cells were resuspended in PBS at 1 × 10^6^ cells/mL and incubated in the presence of mercury orange (10 μΜ) or 2′,7′-Dichlorofluorescin diacetate (DCF-DA) (40 μΜ) respectively, in the dark at 37 °C for 30 min. Then, the cells were washed, resuspended in PBS, and submitted to flow cytometric analysis using a FACSCalibur flow cytometer (Becton Dickinson, Franklin Lakes, NJ, USA) with excitation and emission wavelengths at 488 and 530 nm for ROS and at 488 and 580 nm for GSH. Data were analyzed using ‘BD Cell Quest’ software (Becton Dickinson, Franklin Lakes, NJ, USA). Each experiment was repeated at least three times.

### 2.12. Statistical Analysis

All results were expressed as mean ± SD. Differences were considered significant at *p* < 0.05. One-way ANOVA was performed followed by Tukey’s test for multiple pair-wise comparisons using the SPSS 20.0 software (IBM, Armonk, NY, USA).

## 3. Results

### 3.1. Extract Composition in Bioactive Compounds

The TPC values of the extracts were 267.67, 290.00 and 226.93 mg GAE/g dw, while the TFC values were 65.78, 118.56 and 99.16 mg CE/gr dw for *R. sempervivens*, *R. canina* and *P. coccinea*, respectively (Table 1). 

The results from the qualitative and quantitative assessment of the chemical composition of the extracts as assessed by using HPLC with Diode-Array Detection complemented by UPLC-MS-MS (especially for the quantitative analysis of non-UV sensistive compounds) analysis are depicted in Table 1. The HPLC analysis of the extracts identified polyphenols belonging to different subclasses of flavonoids such as flavanols (e.g., (+)-catechin and (−)-epicatechin), flavonols (e.g., hyperoside, rutin, astragalin, quercitrin, quercetin and kaempferol), flavanonols (e.g., taxifolin), flavanones (e.g., eriodictyol and isosakuranetin), isoflavones (e.g., genistein). In this respect, various polyphenolic acids were also detected such as hydroxybenzoic acids (e.g., gallic acid, syringic acid and protocatechuic acid), hydroxycinnamic acids (e.g., caffeic acid and *p*-coumaric acid) and the chlorogenic acid. Finally, a series of additional molecules were identified such as phloridzin and vanillin polyphenols, quinic acid and the terpenoids betulinic acid and ursolic acid. The latter compounds were detected only in *R. canina* extract.

Specifically, the chemical analysis showed that the *R. canina* extract is particularly rich in polyphenols hyperoside (308.11 μg/g dw), astragalin (172.48 μg/g dw), (+)-catechin (134.75 μg/g dw) and (−)-epicatechin (120.99 μg/g dw) (Table 1). Moreover, *R. canina* extract contained significant concentration of quinic acid (1102.59 μg/g dw) and the terpenoid, ursolic acid (138.23 μg/g dw) (Table 1). In *R. sempervirens* extract, the compounds identified at higher concentrations were quinic acid (389.95 μg/g dw), and the polyphenols (−)-epicatechin (34.01 μg/g dw), (+)-catechin (25.48 μg/g dw), astragalin (9.16 μg/g dw), and hyperoside (8.31 μg/g dw) (Table 1). Finally, *P. coccinea* extract exhibited higher concentrations of hyperoside (170.72 μg/g dw), rutin (25.82 μg/g dw), (−)-epicatechin (10.23 μg/g dw), astragalin (9.13 μg/g dw), (+)-catechin (7.93 μg/g dw), vanillin (7.89 μg/g dw) and syringic acid (6.23 μg/g dw) (Table 1).

### 3.2. Free Radical Scavenging Activity of the Extracts

All three of the extracts exhibited strong free radical scavenging activity. As known, the lower the IC_50_ value, the higher the antioxidant activity. Thus, in the DPPH assay the potency order and the IC_50_ values were: *R. canina* (100 μg/mL) > *R. sempervivens* (130 μg/mL) > *P. coccinea* (500 μg/mL) (Table 2). Similar order of portency was observed in ABTS^•+^ assay; *R. canina* (60 μg/mL) > *R. sempervivens* (85 μg/mL) > *P. coccinea* (140 μg/mL) (Table 2).

**Figure 1:**
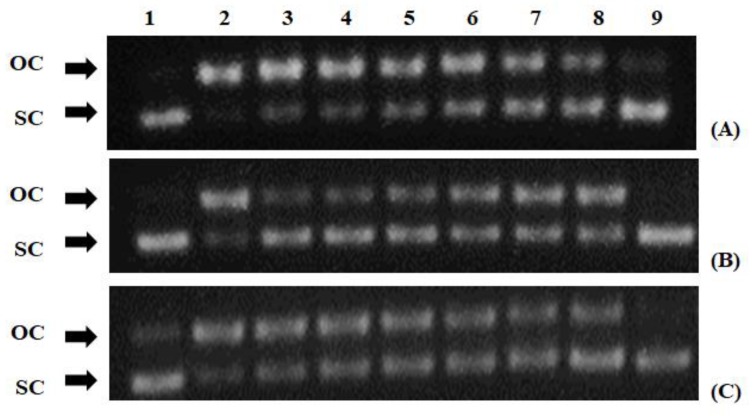
Protective activity of polyphenolic extracts from (**A**) *Pyracantha coccinea*, (**B**) *Rosa sempervivens* and (**C**) *Rosa canina* species against ROO• radical: Lane 1, pBluescript-SK+ plasmid DNA without any treatment; lane 2, plasmid DNA exposed to ROO• radical alone; lanes 3–8 plasmid DNA exposed to ROO• radical in the presence of different concentrations of extracts (*P. coccinea*: 0.063, 0.125, 0.250, 0.500, 1.0 and 1.5 mg/mL; *R. sempervivens*: 2.0, 1.5, 1.0, 0.500, 0.250, 0.125 mg/mL; *R. canina*: 0.063, 0.125, 0.250, 0.500, 1.0 and 1.5 mg/mL); lane 8, plasmid DNA exposed to the maximum tested concentration of each extract alone. OC: open circular; SC: supercoiled.

Finally, all three extracts exhibited protective activity against ROO•-induced DNA plasmid breakage with IC_50_ values and potency order *R. canina* (530 μg/mL) > *R. sempervivens* (570 μg/mL) > *P. coccinea* (950 μg/mL) (Table 2 and Figure 1). 

### 3.3. Effects of Extracts on the Antioxidant Status of Endothelial Cells

**Figure 2:**
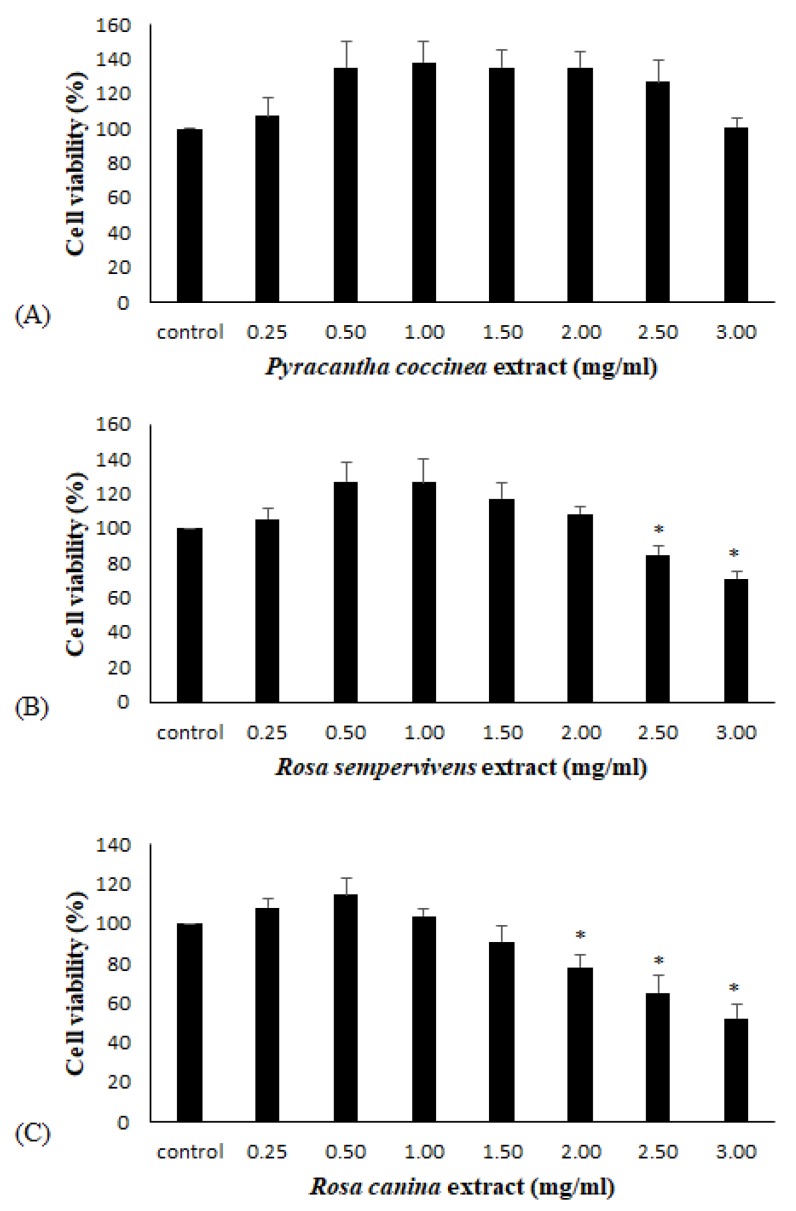
Cell viability following the treatment with polyphenolic extracts from (**A**) *Pyracantha coccinea*, (**B**) *Rosa sempervivens* and (**C**) *Rosa canina* species. The results are presented as the means ± SD of three independent experiments carried out in triplicate. * *p* < 0.05 indicates significant difference from the control value.

To examine the extracts’ antioxidant activity in endothelial cells, flow cytometry analysis was performed. At first, the extract’s effect on cell viability was assessed using the XTT assay, in order to use non-cytotoxic concentrations. The cell viability assay showed that significant cytotoxicity was exhibited at concentrations above 2.5 mg/mL for *R. sempervivens* and 2.0 mg/mL for *R. canina* (Figure 2B,C). None of the concentrations used for *P. coccinea* had cytotoxicity (Figure 2A). Thus, the selected non-cytotoxic concentrations of the extracts in the following assays were up to 1.00 mg/mL.

**Figure 3:**
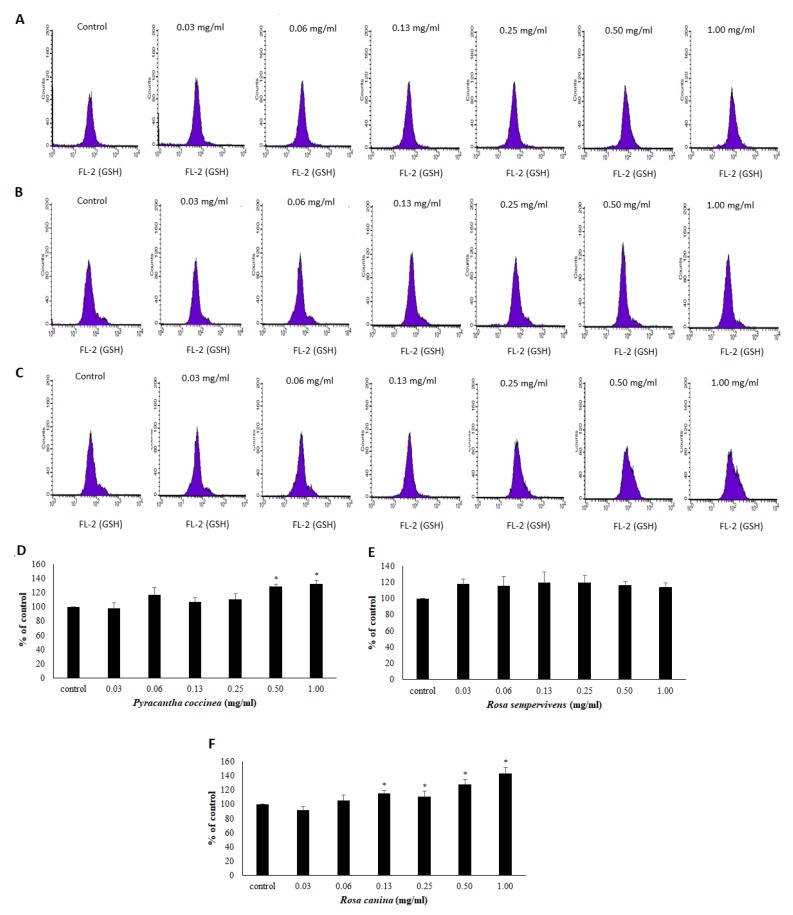
Effects on GSH levels after treatment with *P. coccinea*, *R. sempervivens* and *R. canina* extracts at different concentrations for 24 h in EA.hy926 cells. The histograms of cell counts versus fluorescence of 10,000 cells analyzed by flow cytometry for the detection of GSH levels after treatment with (**A**) *P. coccinea*, (**B**) *R. sempervivens* and (**C**) *R. canina*. FL-2 represents the detection of fluorescence in the FL-2 channel using 488 and 580 nm as the excitation and emission wavelength, respectively. Bar charts indicate the GSH levels as % of control as estimated by the histograms in EA.hy926 cells after treatment with (**D**) *P. coccinea*, (**E**) *R. sempervivens* and (**F**) *R. canina* extracts. All values of bar charts are presented as the mean ± SD of three independent experiments. * *p* < 0.05 indicates significant difference from the control.

The assessment of the extracts’ effects on the antioxidant capacity of endothelial cells was based on the measurement of GSH and ROS levels by flow cytometry analysis. The results demonstrated that *R. canina extract* significantly increased GSH levels by 15.0, 10.4, 28.4 and 43.1% at 0.13, 0.25, 0.50 and 1.00 mg/mL, respectively compared to control (Figure 3C). *P. coccinea* extract also significantly increased GSH levels by 29.2 and 32.3% at 0.50 and 1.00 mg/mL, respectively, compared to control (Figure 3A). However, *R. sempervirens* extract did not affect GSH levels at any of the examined concentrations (Figure 3B).

**Figure 4:**
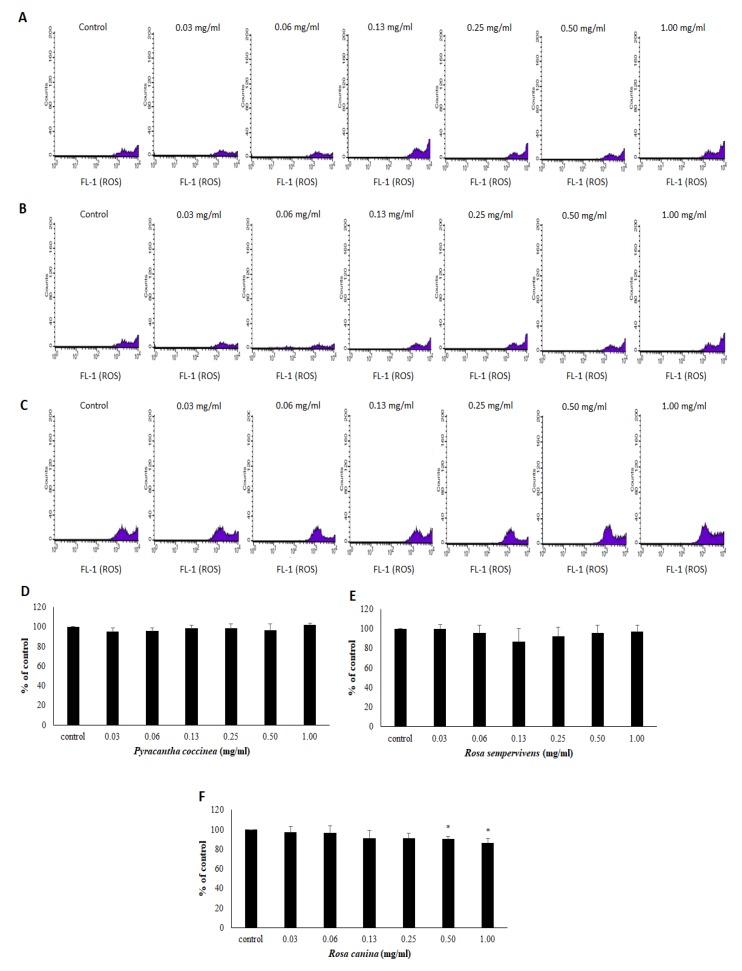
The diagrams show the changes in ROS levels after treatment with *P. coccinea*, *R. sempervivens* and *R. canina* extracts in EA.hy926 cells. The histograms demonstrate the cell counts versus fluorescence of 10,000 cells analyzed by flow cytometry for the detection of ROS levels after treatment with (**A**) *P. coccinea*, (**B**) *R. sempervivens* and (**C**) *R. canina*. FL-1 represents the detection of fluorescence in the FL-1 channel using 488 and 530 nm as the excitation and emission wavelength, respectively. Bar charts indicate the ROS levels as % of control as estimated by the histograms in EA.hy926 cells after treatment with (**D**) *P. coccinea*, (**E**) *R. sempervivens* and (**F**) *R. canina* extracts. All values of bar charts are presented as the mean ± SD of three independent experiments. * *p* < 0.05 indicates significant difference from the control.

The results from the assessment of extracts’ effects on ROS levels are shown in Figure 4. According to the results, only one of the three extracts affected ROS levels. In particular, *R. canina extract* significantly reduced ROS by 9.73 and 13.37% at 0.50 and 1.00 mg/mL, respectively, compared to control (Figure 4C). However, *P. coccinea* and *R. sempervivens* extracts did not significantly affect ROS levels, compared to control (Figure 4A,B). 

## 4. Discussion

The aim of the present study was to assess for the first time the antioxidant effects in endothelial cells of the polyphenolic extracts obtained from the fruits of three wild growing plant species, *R. canina*, *R. sempervirens* and *P. coccinea*. It should be noted that *R. canina* is a well-studied plant species [33], but there is only one study for the polyphenolic composition and the antioxidant activity of *R. semprevirens* fruit extracts [34] and there are only few reports for *P. coccinea* fruit extracts [23].

Initially, the polyphenolic composition of the extracts was analyzed. *R. canina* extract exhibited the highest TPC among the three tested extracts. So, as expected *R. canina* had also the highest TFC. However, although the *R. sempervirens* extract had higher TPC than *P. coccinea*, the TFC of the former was lower than that of the latter. This was probably due to the high content of the *R. sempervirens* extract in polyphenols other than flavonoids. Other studies showed that the TPC values of *R. canina* extracts were from 50 to 500 mg/g dw, and so our results were within this range [35,36]. There has been so far only one study on the polyphenols of *R. sempervirens*, which reported 57.9 mg GAE/g dw for TPC and 0.47 mg CE/g dw for TFC, while our values were 267.67 mg GAE/g dw and 65.78 mg CE/g dw, respectively [34]. Nadpal et al., [34] have used different solvents (i.e., methanol and water) for isolating polyphenolic ectracts from *R. sempervirens.* Their results showed that water extraction yielded higher TPC and TFC values compared to methanol extraction [34]. Moreover, another study demonstrated that the TPC values were different between *R. canina* extracts isolated from plants grown in different locations [36]. In a previous study, it was also shown that ethanol extracts of *R. canina* had higher TPC values than water extracts [36]. Bhave et al., [37] have also shown that the content of biologically active compounds in Rosa species depended on specific genotypes. In addition, extracts from more ripened fruits of *R. canina* have been reported to have higher TPC compared to less ripened fruits [38]. Thus, the differences between our results and those from other studies could be atrributed to different factors such as differences in the analytical methods, different extraction methods, genetic and environmental factors and the maturity stage of fruits and harvesting time [37,38,39]. 

The HPLC analysis showed that the *R. canina* extract was especially rich in the polyphenols hyperoside, astragalin, rutin, (+)-catechin and (−)-epicatechin as well as in the terpenoid ursolic acid and a poly-hydroxylated organic acid organic acid, which is the quinic acid. The presence of these compounds have also been reported in previous studies [33,40]. Moreover, several other polyphenols have been identified in trace amounts in *R. canina* extracts such as ellagic acid, salicylic acid, vanillic acid, ferulic acid and caffeic acid (not found by us) [33]. The *R. sempervirens* extract had higher amounts of quinic acid, (+)-catechin, (−)-epicatechin, astragalin, and hyperoside. Like our findings, Nadpal et al. (2018) [34] have also identified gallic acid, quercitrin, quercetin, hyperoside and (+)-catechin in *R. sempervirens* extract, although not at the same concentrations as our samples. However, Nadpal et al. (2018) [34] have also reported ellagic acid, protocatechuic acid (not found in our extract), ferulic acid and kaempferol-3-*O*-glucoside. In general, our study is the first that identified quinic acid, (−)-epicatechin, rutin, astragalin, eriodictyol, and genistein in *R. sempervirens* extract. The *P. coccinea* extract contained higher concentrations of hyperoside, rutin, (−)-epicatechin, (+)-catechin, astragalin, vanillin, syringic acid and chlorogenic acid. As expected, the polyphenolic profiles of the two extracts from the *Rosa* genus (i.e., *R. canina* and *R. sempervirens*) had more similaritities with each other than with the extract from *P. coccinea* of *Pyracantha* genus. 

All three extracts exhibited strong free radical scavenging activity in DPPH and ABTS•^+^ assays. The highest potency of the *R. canina* extract in both assays was in accordance with its highest TPC value. Some studies have reported IC_50_ values in DPPH assay for *R. canina* extracts, which were similar to ours, but other studies have found values that were significantly different from ours [18,40]. Previous studies have also shown that polyphenolics were the most important compounds of *R. canina* extracts for the DPPH radical scavenging activity [40]. *R. sempervirens* extract having higher TPC than *P. coccinea* extract also exhibited greater free radical scavenging activity. Nadpal et al. (2018) [34] have reported 28 μg/mL as IC_50_ value in DPPH assay for a *R. sempervirens* extract, while our value was 130 μg/mL. The differences between our values and those from other studies are probably, as mentioned above, due to different factors which lead to differences in the polyphenolic composition of the extracts.

Moreover, all three extracts exerted protective activity against ROO•-induced DNA damage. Like in DPPH and ABTS•^+^ assays, the potency order in this assay followed the order of polyphenolic concentration, that is, *R. canina* > *R. sempervirens* > *P. coccinea*. Therefore, once again, the polyphenolic concentration seemed to play a crucial role in the protective activity from ROO•-induced DNA damage. Among the polyphenols identified in the extracts, (+)-catechin, (−)-epicatechin, rutin, vanillin, astragalin, phloridzin and gallic acid have been reported to scavenge ROO• radical [41,42,43,44,45,46,47]. As far as we know, protective activity of *R. canina*, *R. sempervirens* and *P. coccinea* extracts from free radical-induced DNA damage was examined for the first time in this study. Thus, based on these results, extracts from the tested plant species may be used for protection against diseases caused by ROS-induced DNA damage.

Since the tested extracts showed strong free radical scavenging activity, their antioxidant effects were also investigated at noncytotoxic concentrations in endothelial cells. Thus, the extracts’ ability to increase GSH, one of the most important antioxidant molecules within cells, was assessed by flow cytometry [48]. In agreement with the free radical scavenging assays, *R. canina* extract exhibited the greatest capacity to increase GSH in the cells. However, it should be noted that when humans consumed rose hip powder from *R. canina*, there were no effects on the activity of enzymes related to GSH metabolism in erythrocytes [49]. Treatment of cells with *P. coccinea* extract also increased significantly GSH levels in EA.hy926 cells. However, *R. sempervirens* extract treatment had no effect on GSH levels. This finding was intriguing, since *R. sempervirens* extract contained more polyphenols than *P. coccinea* extract, while *R. sempervirens* extract’s polyphenolic profile was quite similar to that of *R. canina*. This contradiction could be explained by examining the polyphenols that were found at a greater concentration in *P. coccinea* extract than in *R. sempervirens*. For example, hyperoside and rutin found at higher concentration in *R. canina* and *P. coccinea* extracts compared to *R. sempervirens* have been reported to increase GSH levels in endothelial cells [50,51]. The observed increase in GSH levels induced by *R. canina* and *P. coccinea* extract treatment is important, since GSH apart from its antioxidant role is a crucial regulator of cell signaling in endothelial cells [52,53]. 

Regarding extracts’ effects on ROS levels in endothelial cells, only *R. canina* extract treatment exerted a significant decrease. This result was in accordance with *R. canina* extract’s highest antioxidant potency exhibited in all the other assays. Moreover, *R. canina* extract-induced decrease in ROS levels was attributed, at least in part, to extract’s capacity to increase antioxidant defense mechanisms such as GSH. Moreover, a *R. canina* extract has been shown to inhibit an H_2_O_2_-induced increase in ROS in colon cancer cells [54]. Furthermore, some polyphenols such as (+)-catechin, (−)-epicatechin and ursolic acid identified at higher concentrations in *R. canina* than in the other two tested extracts have been demonstrated to decrease ROS in endothelial EA.hy926 and human umbilical vein endothelial cells (HUVEC) cells [55,56,57].

In conclusion, the results of the present study provided new information concerning the polyphenolic composition of the tested extracts, especially those of *R. sempervirens* and *P. coccinea* fruit extracts. Moreover, all three tested extracts were demonstrated for the first time to protect against ROS-induced DNA damage, which thus suggests their possible use for prevention of relative diseases. In addition, the extracts from *R. canina* and *P. coccinea* were shown to increase GSH levels, the most important antioxidant molecule in endothelial cells. This finding suggests that these extracts may be used for the development of food supplements or biofunctional foods preventing diseases caused by oxidative damage to endothelium such as cardiovascular. Interestingly, previous *in vivo* studies have reported that administration of rose hip powder to humans or experimental animals could reduce the risk for cardiovascular diseases [22,58]. Of course, further studies are needed in order to investigate further the molecular mechanisms through which these protective activities are exerted. Moreover, since the environmental variability between different years may affect the chemical composition of the plant extracts and consequently their bioactivities, it should also be examined how the tested activities are changing from one year to the next.

## Figures and Tables

**Table 1:** Bioactive compounds of extracts from dried fruits *R. canina*, *R. semprevirens* and *P. coccinea*.

Compound	*R. canina* ^a^	*R. semprevirens* ^a^	*P. coccinea* ^a^
Quinic acid	1102.59 ± 38.91	389.95 ± 10.29	ND
(+)-Catechin	134.75 ± 1.02	25.48 ± 0.68	7.93 ± 0.31
Gallic acid	2.21 ± 0.09	0.44 ± 0.03	ND
Protocatechuic acid	2.09 ± 0.06	ND	ND
Syringic acid	ND	ND	6.23 ± 0.17
Caffeic acid	ND	ND	1.49 ± 0.08
(−)-Epicatechin	120.99 ± 1.18	34.01 ± 0.51	10.23 ± 1.10
Hyperoside	308.11 ± 7.10	8.31 ± 0.19	170.72 ± 3.49
Rutin	25.64 ± 0.48	2.62 ± 0.14	25.82 ± 0.98
Chlorogenic acid	ND	ND	4.82 ± 0.06
Taxifolin	ND	ND	0.09 ± 0.02
*p*-Coumaric acid	2.44 ± 0.07	ND	ND
Vanillin	ND	ND	7.89 ± 0.06
Astragalin	172.48 ± 7.48	9.16 ± 0.10	9.13 ± 0.05
Phloridzin	3.41 ± 0.10	ND	ND
Eriodictyol	ND	0.05 ± 0.01	0.06 ± 0.01
Quercitrin	ND	0.44 ± 0.07	ND
Quercetin	0.67 ± 0.05	0.19 ± 0.02	0.06 ± 0.01
Genistein	ND	0.03 ± 0.01	ND
Kaempferol	0.46 ± 0.02	ND	0.05 ± 0.01
Isorhamnetin	ND	ND	ND
Isosakuranetin	ND	ND	0.03 ± 0.01
Betulinic acid	0.47 ± 0.03	ND	ND
Ursolic acid	138.23 ± 4.44	ND	ND
TPC ^b^	290.00 ± 2.10 ^d^	267.67 ± 1.78 ^e^	226.93 ± 1.11 ^f^
TFC ^c^	118.56 ± 1.69 ^g^	65.78 ± 0.93 ^h^	99.16 ± 1.22 ^i^

ND: not detected. ^a^ Values are expressed as μg/g of dried weight of extract and are the mean ± SD from three measurements. ^b^ TPC: Total Polyphenolic Content, expressed as mg of gallic acid equivalent/g dried weight extract. ^c^ TFC: Total Flavonoid Content, expressed as mg of catechin equivalent/g dried weight extract. ^d,e,f^ Values with different superscript letters are significantly different between them (*p* < 0.05). ^g,h,i^ Values with different superscript letters are significantly different between them (*p* < 0.05).

**Table 2:** Free radical scavenging activity against DPPH and ABTS radicals, protective activity against peroxyl radical (ROO•)-induced DNA damage of the extracts.

Plant Extracts	IC_50_ (μg/mL)		
	DPPH ^a^	ABTS ^a^	ROO ^b^
*Rosa sempervivens* (fruit)	130 ± 7.8 ^a,^*	85 ± 10.0 ^d,^*	570 ± 51.3 ^g,^*
*Rosa canina* (fruit)	100 ± 7.0 ^b,^*	60 ± 6.6 ^e,^*	530 ± 68.9 ^g,^*
*Pyracantha coccinea* (fruit)	500 ± 40.0 ^c,^*	140 ± 4.2 ^f,^*	950 ± 47.5 ^h,^*

^a^ Values are the mean ± SD of at least two separate triplicate experiments. ^b^ Values are the mean ± SD from three independent experiments. * *p* < 0.05, indicates significant difference from the control values. ^a,b,c^ Values with different superscript letters are significantly different between them (*p* < 0.05). ^d,e,f^ Values with different superscript letters are significantly different between them (*p* < 0.05). ^g,h^ Values with different superscript letters are significantly different between them (*p* < 0.05).
